# Transcriptome Analysis of the Thymus in Short-Term Calorie-Restricted Mice Using RNA-seq

**DOI:** 10.1155/2018/7647980

**Published:** 2018-02-04

**Authors:** Zehra Omeroğlu Ulu, Salih Ulu, Soner Dogan, Bilge Guvenc Tuna, Nehir Ozdemir Ozgenturk

**Affiliations:** ^1^Faculty of Art and Science, Molecular Biology and Genetics, Yildiz Technical University, Istanbul, Turkey; ^2^Department of Medical Biology, School of Medicine, Yeditepe University, Istanbul, Turkey; ^3^Department of Medical Biophysic, School of Medicine, Yeditepe University, Istanbul, Turkey

## Abstract

Calorie restriction (CR), which is a factor that expands lifespan and an important player in immune response, is an effective protective method against cancer development. Thymus, which plays a critical role in the development of the immune system, reacts to nutrition deficiency quickly. RNA-seq-based transcriptome sequencing was performed to thymus tissues of MMTV-TGF-*α* mice subjected to ad libitum (AL), chronic calorie restriction (CCR), and intermittent calorie restriction (ICR) diets in this study. Three cDNA libraries were sequenced using Illumina HiSeq™ 4000 to produce 100 base pair-end reads. On average, 105 million clean reads were mapped and in total 6091 significantly differentially expressed genes (DEGs) were identified (*p* < 0.05). These DEGs were clustered into Gene Ontology (GO) categories. The expression pattern revealed by RNA-seq was validated by quantitative real-time PCR (qPCR) analysis of four important genes, which are leptin, ghrelin, Igf1, and adinopectin. RNA-seq data has been deposited in NCBI Gene Expression Omnibus (GEO) database (GSE95371). We report the use of RNA sequencing to find DEGs that are affected by different feeding regimes in the thymus.

## 1. Introduction

Calorie restriction (CR) is the reduction in calorie intake without inducing malnutrition [1, 2]. The two main types of calorie restriction are chronic calorie restriction (CCR) and intermittent calorie restriction (ICR). ICR refers to the application of calorie restriction in periods “on” and “off” [3–6]. Researchers have reported that CR is a more highly effective experimental manipulation for suppressing tumor development [7, 8], suppressing autoimmunity [9, 10], and extending lifespan [11] than fed ad libitum (AL) diet, in rodents [12]. Moreover, in genetically engineered animal models, several studies have shown that ICR is more effective for prevention of cancer development compared to CCR [4, 13–16], while other studies have found that ICR is less effective for cancer prevention than CCR [17–22].

The thymus, which plays an important role in the development of the immune system, is a primary lymphoid organ and a place of T-cell differentiation and maturation [23, 24]. The thymus and other lymphoid organs react to nutrition deficiency more rapidly than most of the other organs [25, 26]. Several studies show that CR potentiates thymic function [2] and can regulate thymic adiposity [27], since CR specifically inhibits the adipogenic transcription in the aging thymus [28].

Breast cancer is the most frequent cancer in women and causes mainly the death of millions of women each year. Animal studies have shown that calorie restriction prevents mammary tumor development [29–32]. MMTV-TGF-*α* transgenic mice have a particular value in age-related mammary tumor (MT) development studies. These mice have been reported to develop MT in their second year of life [33] and overexpress TGF-*α*, epidermal growth factor, which plays a critical role in the development of the human breast cancer [34–38].

RNA sequencing (RNA-seq) is one of the applications of the next generation sequencing (NGS) technologies, with which gene expression can be measured [39–42]. It is a sensitive, fast, and efficient method for gene discovery in organisms [43, 44]. Moreover, RNA-seq has been successfully used for annotation, transcript profiling, detecting gene fusions, single-nucleotide polymorphism (SNP) discovery, and detecting alternatively spliced RNA forms [40, 45–49].

In the present study, we used RNA-seq technology to perform a comparative transcriptome analysis of the MMTV-TGF-*α* female mouse thymus tissues. The mice were subjected to AL, CCR (85% of AL-fed mice), and ICR (3 weeks AL fed, 1 week 40% of AL-fed mice) diets from 10 weeks of age to 17 weeks of age or 18 weeks of age. The aim of this study is to determine the differences in the gene expression profiles of the thymus tissue due to different feeding regimes.

## 2. Materials and Methods

### 2.1. Animals and Experimental Design

In this experiment, MMTV-TGF-*α* (C57/BL6) female mice were used. These mice overexpress human TGF-*α*, a part of epidermal growth factor receptor (EGFR)/ErbB cascade which is known to play a role in the development of human breast cancers [34–38]. Mice colonies were maintained using a breeding protocol and genotyping assay at Yeditepe University Animal Facility, as previously described [13]. At 10 weeks of age, female MMTV-TGF-*α* mice were assigned to one of the following dietary groups: ad libitum (AL), chronic caloric restricted (CCR), and intermittent caloric restricted (ICR). The CCR group received 85% of the daily food consumption of AL mice, in other words, 15% caloric restriction were applied to them. The ICR group was fed AL for 3 weeks, and then for the following week, 60% caloric restriction compared to AL was applied for one week. Mice diets (Altromin TPF1414) were purchased from Kobay AS (Ankara, Turkey). All mice had free access to water. Body weights were measured weekly, and food intakes were determined daily, for all mice. The health statuses of the animals were checked by an expert veterinarian on a regular basis, at least once a week. Mice were euthanized after overnight fasting, at the age of 17 or 18 weeks old. Mice in the ICR group were euthanized after three weeks of AL feeding. Thymus tissues were collected in liquid nitrogen and stored at minus 80°C, until used.

### 2.2. RNA Extraction, Library Construction, and Sequencing

Total RNA was extracted from the thymus tissue samples of three different individual mice in each different diet group, using Trizol extraction method (Invitrogen, Carlsbad, CA, USA) according to the manufacturer's protocol, then further purified with RNeasy columns (Qiagen). The concentration of each RNA sample was measured with BioSpec-nano UV-VIS specthrophometer (Shimadzu, Kyoto, Japan). The integrity of the RNA was assessed by the Agilent 2100 Bioanalyzer system (Agilent Technologies, Santa Clara, CA). The mRNA sequencing libraries for RNA-seq were constructed with the TruSeq RNA Sample Prep Kit (Illumina, San Diego, CA), according to the manufacturer's instructions. Pair-end (2 × 100 bp) sequencing was performed using an Illumina HiSeq 4000 Sequencing System (Illumina) at Beijing Genomics Institute (BGI). RNA-seq data has been deposited in NCBI Gene Expression Omnibus (GEO) database under accession number GSE95371.

### 2.3. RNA-seq Data Processing

Raw pair-end reads were subjected to quality control, and clean reads were filtered with FASTX-Toolkit (http://hannonlab.cshl.edu/fastx_toolkit/index.html). Clean reads were mapped to GRCm38 mouse assembly in the Ensemble database using Tophat 2.0.13 [50]. Gene expression levels were quantified by Cufflinks 2.2.1 and normalized by the fragments per kilobase of transcript per million fragments mapped method (FPKM). The differentially expressed genes (DEGs) were identified with Cuffdiff, a part of Cufflinks package [51]. The DEGs were filtered employing the false discovery rate (FDR) correction, Fisher's exact test, and a fold-change method, exhibiting a corrected *p* value not greater than 0.05 (*p* value < 0.05) along with a fold change value not less than 2.0. All of the data produced by a Cuffdiff analysis was visualized and integrated with *R* [52].

Gene Ontology (GO) is an international, standardized, gene-function classification system. GO enrichment analysis identifies all of the GO terms that are significantly enriched in DEGs compared with the genome background and filters the DEGs that correspond to biological functions. According to this method, all DEGs have been mapped to GO terms in the database (http://www.geneontology.org/); gene numbers have been calculated for every term, using a hypergeometric distribution compared with the genome background [43]. We mapped all the DEGs obtained from these libraries (*p* value < 0.05) to GO database, to classify for enriched GO terms.

### 2.4. RNA-seq Data Validation by qPCR

After the analysis of RNA-seq data, four of the important genes for calorie restriction studies, leptin (Lep), ghrelin (Ghr), insulin-like growth factor 1 (Igf1), and adinopectin (Adipoq), were selected and their differential expression results were validated by using quantitative real-time PCR (qPCR). All qPCR reactions were run in replicates using GM SYBR Green qPCR kit and conducted on the LightCycler Nano real-time system (Roche, Switzerland). The PCR conditions were as follows: denaturation at 95°C for 2 min followed by 45 cycles of amplification (95°C for 20 s, 58°C for 30 s, and 72°C for 45 s). Primer sequences can be found in Table 1. The 2^−ΔCT^ method was used to calculate relative gene expression levels in each sample, and Gapdh was used as an internal control.

## 3. Results

### 3.1. Summary of RNA Isolation, RNA-seq Libraries, and Mapping

Nine total RNA isolations were done, and three sequencing libraries were constructed from thymus tissues of MMTV-TGF-*α* mice in the AL, CCR, and ICR diet groups for RNA-seq. Three libraries were subsequently sequenced on an Illumina HiSeq 4000 Sequencing System (Illumina) generated about 127 million raw reads. After filtering adapter sequences, contamination, and low-quality sequences, a total of 105 million clean reads were finally produced (Table 2).

Clean reads were mapped to GRCm38 mouse assembly in the Ensemble database with TopHat. As a result, mapping ratios (mapped reads/all reads) of 91.9%–94.9% were attained for all the three sequencing libraries.

### 3.2. Differentially Expressed Genes (DEGs) and Gene Ontology (GO) Analysis

To detect transcriptomic changes in gene expression, gene expression levels were computed by Cufflinks 2.2.1 and normalized to FPKM values. 44,426 detected genes were quantified with corrected FPKM values. According to the results, the number of isoforms, TSS (transcription start site), CDS (coding sequences), and promoters were quantified 138,066, 80,392, 48,900, and 133,278 in three sequencing libraries, respectively. Corrected FPKM values were subjected to analysis of DEGs; a total of 6091 significantly differentially expressed genes were identified in three different diet groups, by using corrected *p* value < 0.05 as the filter (Table 3).

The 2821, 2825, and 445 significantly differentially expressed genes were detected between the diet groups AL-CCR, CCR-ICR, and AL-ICR, respectively (*p* < 0.05). The numbers of significantly differentially expressed genes (DEGs) and numbers of isoforms, TSS, and CDS between the diet groups are shown in Table 3. According to these results, 916, 1877, and 200 genes were upregulated and 1905, 948, and 245 genes were downregulated in DEGs between the diet groups AL and CCR, CCR and ICR, and AL and ICR, respectively.

**Figure 1:**
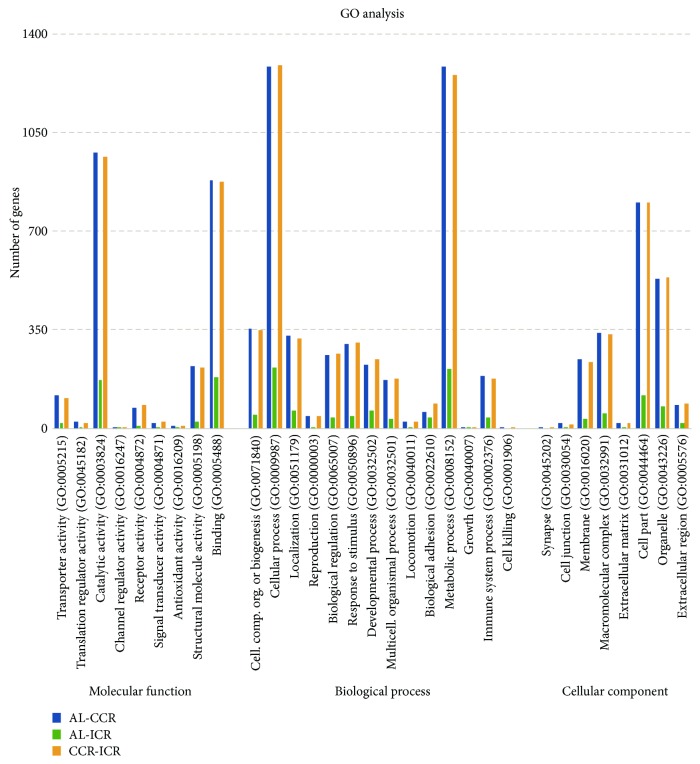
Clasifications and numbers of DEGs into GO terms (AL-CCR: DEGs between AL and CCR; AL-ICR: DEGs between AL and ICR; and CCR-ICR: DEGs between CCR and ICR).

For better understanding of gene function, DEGs obtained from three libraries were further subjected to Gene Ontology (GO) functional enrichment analysis, which provided biological terms to identify gene products in three perspectives: cellular components, biological processes, and molecular functions. DEGs obtained between the AL and CCR, AL and ICR, and CCR and ICR diet groups (*p* < 0.05) were classified according to three main categories of GO terms via http://www.geneontology.org/, as shown in Figure 1.

The assigned functions of DEGs covered a broad range of GO categories. In the molecular function category, catalytic activity (GO:0003824) (978 unigenes, 34.6%) and binding (GO:0005488) (882 unigenes, 31.2%) in significant 2821 DEGs between AL and CCR (AL-CCR), catalytic activity (GO:0003824) (170 unigenes, 38%) and binding (GO:0005488) (183 unigenes, 41.1%) in significant 445 DEGs between AL and ICR (AL-ICR), and catalytic activity (GO:0003824) (966 unigenes, 34.1%) and binding (GO:0005488) (875 unigenes, 31%) in significant 2825 DEGs between CCR and ICR (CCR-ICR) were prominently represented.

GO results showed that in the top two abundant GO terms in biological process, categories were cellular process (GO:0009987) and metabolic process (GO:0008152). In cellular process (GO:0009987), 1283 unigenes (45.4%), 216 unigenes (48.5%), and 1288 unigenes (45.5%) were involved in significant DEGs AL-CCR, AL-ICR, and CCR-ICR, respectively. Also, in metabolic process (GO:0008152), 1285 unigenes (45.5%), 212 unigenes (47.6%), and 1255 (44.4%) were involved in significant DEGs AL-CCR, AL-ICR, and CCR-ICR, respectively.

In the category of cellular component, 802, 119, and 802 unigenes were located in the cell parts, and 531, 78, and 537 unigenes were located in the organelle parts.

**Figure 2:**
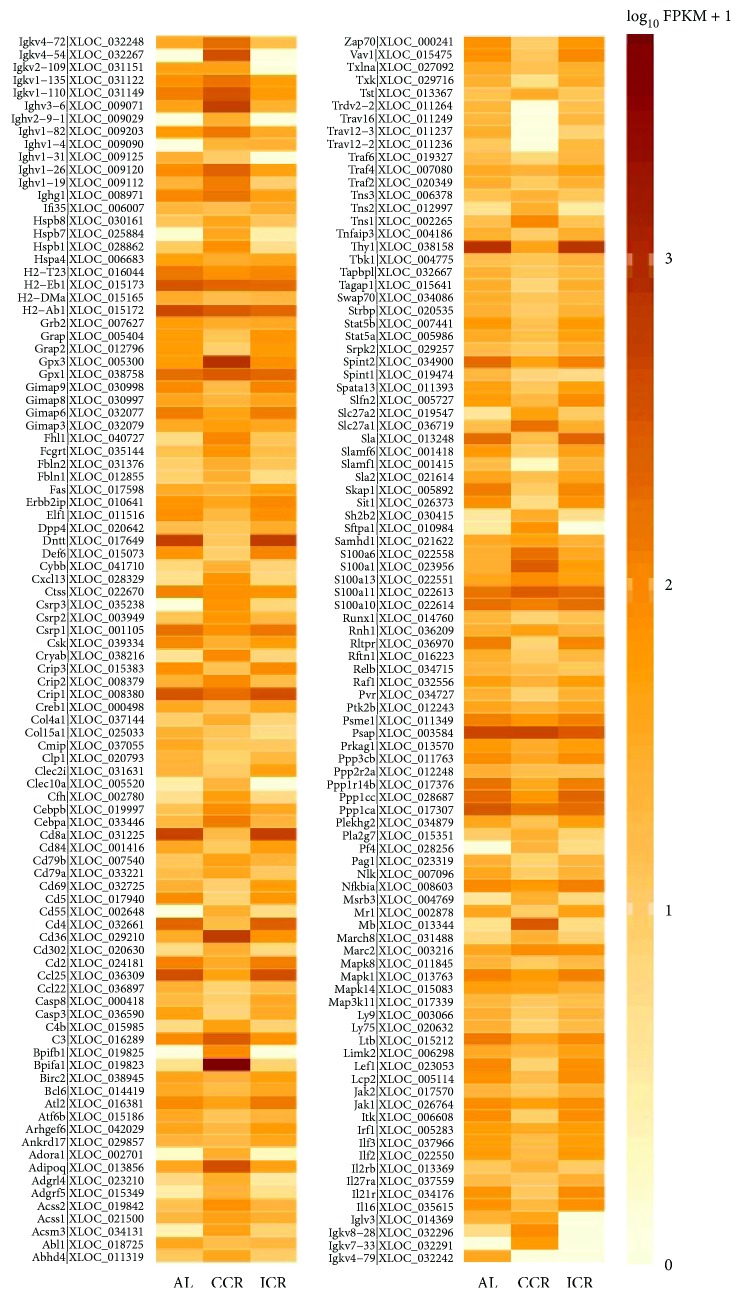
Heat map showing the expression profiles of significantly differentially expressed genes between the AL, CCR, and ICR diet groups involved in immune response processes (GO: 0002376).

Besides, the expression of genes grouped as “immune system process” GO term (0002376) based on analyzed transcriptome reveals that 188 of 2821, 36 of 445, and 176 of 2825 genes were differentially expressed between AL-CCR, AL-ICR, and CCR-ICR diet groups, respectively. These DEGs were shown in Figure 2 according to the three different diet groups.

### 3.3. Validation of Gene Expression

**Figure 3:**
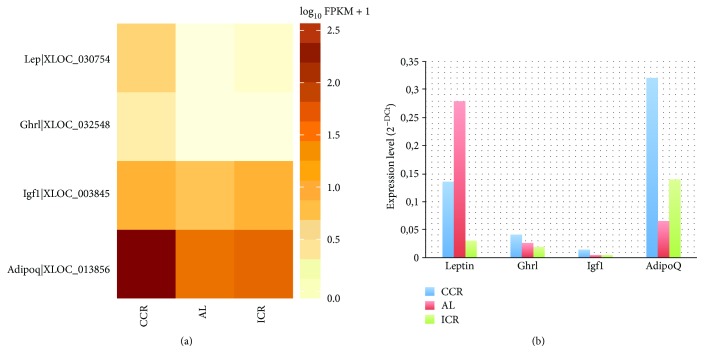
(a) Heat map showing the expression profiles of leptin, ghrelin, Igf1, and adiponectin genes in the CCR, AL, and ICR groups revealed by RNA-seq. (b) qPCR validation showing the expression levels of leptin, ghrelin, Igf1, and adiponectin genes in the CCR, AL, and ICR groups.

To validate the results of differentially expressed genes in transcriptome sequencing, leptin (Lep), ghrelin (Ghr), insulin-like growth factor 1 (Igf1), and adinopectin (Adipoq) genes were selected because they were differentially expressed in each diet group. Also, important roles of these genes in adipogenesis, nutrition metabolism, and tumor development have been reported [15, 53–56]. The results of RNA-seq (Figure 3(a)) and qPCR (Figure 3(b)) for four differentially expressed genes were shown in Figure 3. According to the results of RNA-seq, Lep, Ghr, and Adipoq were upregulated in the CCR diet group compared with the ICR and AL diet groups but Igf1 was upregulated in the CCR and ICR diet groups compared with the AL diet group (Figure 3(a)). According to the qPCR results, Ghr, Igf1, and Adipoq were upregulated in the CCR diet group compared with the ICR and AL diet groups but Lep was upregulated in the AL diet group compared with other diet groups (Figure 3(b)).

## 4. Discussion

Calorie restriction (CR) is a strong metabolic intervention that induces a state of chronic negative energy balance and robustly expands mean and maximal lifespan in experimental animals [57, 58]. There are many studies from animal models that suggest that calorie restriction has a significant impact on various arms of the immune system. Most of the reports suggest that CR improves many parameters of immune responses [2, 28, 56]. There are two major calorie restriction applications: chronic calorie restriction (CCR) and intermittent calorie restriction (ICR). Although CCR method is commonly applied in most studies, there are a limited number of studies that have been reported for ICR. In many experimental animal studies, ICR and CCR have been reported to have anticancer effects. Some of the studies show that compared to the AL group, CCR is more effective, while the others showed that ICR is more effective [59].

The thymus is one of the major lymphoid organs in the immune system, and several studies have reported that calorie restriction (CR) potentiates thymic function [2, 28, 60]. RNA were isolated from thymus tissue of ad libitum, chronic calorie restriction, and intermittent calorie restriction diet MMTV-TGF-*α* mice from 10 weeks of age to 17 weeks of age or 18 weeks of age. RNA-seq resulted in an average of ~127 million raw reads, 100 bp reads per sample with average of ~95 million mapped reads. 6091 significantly differentially expressed genes (DEGs) were obtained between three diet groups. The results of DEGs were annotated into molecular function, cellular component, and biological process GO terms.

According to the DEG analysis, there are 2821, 2825, and 445 significantly differentially expressed genes between the diet groups AL-CCR, CCR-ICR, and AL-ICR, respectively. These results show that calorie restriction and/or the types of calorie consumption has a great effect on gene expression in the thymus. Compared to the AL group, although only 15% of calorie restriction was applied to the CCR diet group, 2821 DEGs were shown between the AL and CCR diet groups. Despite the fact that, compared to the AL diet group, only one-week long calorie restriction (60%) was applied to the ICR group, 445 significantly DEGs were determined between the AL and ICR groups.

The studies have reported that adinopectin (Adipoq), leptin (Lep), ghrelin (Ghr), and insulin-like growth factor (Igf1) hormones have an important role in the cancer development and immunity [15, 54, 56]. Adinopectin is a significant hormone to initiate insulin sensitivity; its levels rise during CR [61, 62]. Leptin decreases the level of stress hormones and rises thyroid activity and thyroid-hormone levels [63]. Because CR upregulates stress hormones and downregulates thyroid hormones, leptin levels decline in CR [63, 64]. Ghrelin also regulates immune function by reducing proinflammatory cytokines [56]. In the present study, increased Igf1 levels were detected in the CCR and ICR diet groups, compared to the AL group. Although many studies have reported reduced level of Igf1 with calorie restriction [65–67], there are other reports that show either no change or increase in Igf1 levels with calorie restriction [68, 69]. In this context, current results support our previous findings which was done using the same mouse model [70]. In our previous study, we also reported increased levels of Igf1 and IGFBP3 protein expressions in mammary fat pad tissue of mice of the CCR and ICR diet groups, compared to the AL group at 37 weeks of age [70]. In addition, Igf1 gene expression levels were similar among all diet groups. It should also be noticed that 18 weeks of age is considered to be young for a mouse model. Therefore, it is not unexpected to see higher Igf1 gene expression levels in the calorie-restricted group which needs more growth factors. With respect to our RNA-seq and qPCR results, adinopectin gene expression level increases in the CCR diet group. According to RNA-seq results, leptin gene expression level is highest in the CCR diet group, but according to the qPCR results, it has highest expression level in the AL diet group. Ghrelin gene expression was only observed in the CCR group; this result indicates CCR effects developing immune function positively.

The current studies suggest that CR improves many parameters of immune responses [2, 28, 56, 71, 72], such as responses of T-cells to mitogens, natural killer cell activity, and the ability of mononuclear cells to produce proinflammatory cytokines. According to the GO analysis results, the number of immune response of DEGs was higher in between the AL-CCR and CCR-ICR diet groups, than between the AL-ICR groups. This shows that the expression of immune system genes was regulated up or downregulated by chronic diet.

Results from present study indicate that RNA-seq is a powerful tool to analyze transcriptomes, to study gene expression profiles and to compare distinct stages of different conditions. According to our findings, differences of gene expression have occurred in AL, CCR, and ICR diet types and these results will provide new clues for caloric restriction, immune system, and cancer development studies.

## Figures and Tables

**Table 1:** The information of the primer pairs used for the analysis of gene expression levels by qPCR.

Gene name	Primer	Product size (bp)
Leptin	5′GGT TGT CCA GGG TTG ATC TC 3′	110 bp
	5′GTG GGA GAC AGG GTT CTA CT3′	
Ghrl	5′GCT GTC TTC AGG CAC CAT CT3′	113 bp
	5′TTC TCT GCT GGG CTT TCT GG5′	
Igf1	5′CAA GTC CAG AGA GGA AGC TAT G3′	155 bp
	5′CCG AGA GGT GGA GTG ATT TG3′	
AdipoQ	5′GCA CGA GGG ATG CTA CTG TT3′	127 bp
	5′CAC AAG TTC CCT TGG GTG GA3′	
Gapdh	5′ACT CCA CTC ACG GCA AAT TC3′	150 bp
	5′CAG TAG ACT CCA CGA CAT ACT C3′	

**Table 2:** The statistical results for the AL, CCR, and ICR diet groups' libraries.

Diet groups	Raw reads	Clean reads	Read length (bp)	Clean bases	GC (%)
AL	39,760,624	34,926,546	100	3,492,654,600	49.63
CCR	39,760,624	34,991,272	100	3,499,127,200	48.4
ICR	47,826,710	35,113,458	100	3,511,345,800	47.91

**Table 3:** Number of genes differentially expressed between diet groups.

Diet groups	AL-CCR	CCR-ICR	AL-ICR
Number of significantly DEGs	2821	2825	445
Number of isoforms	1686	1637	331
Number of TSS	2391	2314	461
Number of CDS	1739	1614	316
